# An improved sequence based prediction protocol for DNA-binding proteins using SVM and comprehensive feature analysis

**DOI:** 10.1186/1471-2105-14-90

**Published:** 2013-03-09

**Authors:** Chuanxin Zou, Jiayu Gong, Honglin Li

**Affiliations:** 1 Shanghai Key Laboratory of New Drug Design, State Key Laboratory of Bioreactor Engineering, School of Pharmacy, East China University of Science and Technology, Shanghai, 200237, China

## Abstract

**Background:**

DNA-binding proteins (DNA-BPs) play a pivotal role in both eukaryotic and prokaryotic proteomes. There have been several computational methods proposed in the literature to deal with the DNA-BPs, many informative features and properties were used and proved to have significant impact on this problem. However the ultimate goal of Bioinformatics is to be able to predict the DNA-BPs directly from primary sequence.

**Results:**

In this work, the focus is how to transform these informative features into uniform numeric representation appropriately and improve the prediction accuracy of our SVM-based classifier for DNA-BPs. A systematic representation of some selected features known to perform well is investigated here. Firstly, four kinds of protein properties are obtained and used to describe the protein sequence. Secondly, three different feature transformation methods (OCTD, AC and SAA) are adopted to obtain numeric feature vectors from three main levels: Global, Nonlocal and Local of protein sequence and their performances are exhaustively investigated. At last, the mRMR-IFS feature selection method and ensemble learning approach are utilized to determine the best prediction model. Besides, the optimal features selected by mRMR-IFS are illustrated based on the observed results which may provide useful insights for revealing the mechanisms of protein-DNA interactions. For five-fold cross-validation over the DNAdset and DNAaset, we obtained an overall accuracy of 0.940 and 0.811, MCC of 0.881 and 0.614 respectively.

**Conclusions:**

The good results suggest that it can efficiently develop an entirely sequence-based protocol that transforms and integrates informative features from different scales used by SVM to predict DNA-BPs accurately. Moreover, a novel systematic framework for sequence descriptor-based protein function prediction is proposed here.

## Background

DNA binding proteins (DNA-BPs) that interact with DNA are pivotal to the cell function such as DNA replication, transcription, packaging recombination and other fundamental activities associated with DNA. DNA-BPs represent a broad category of proteins, known to be highly diverse in sequence, structure and function. Structurally, they have been divided into eight structural/functional groups, which were further classified into 54 structural families [1]. Functionally, protein-DNA interactions play various roles across the entire genome as previously mentioned [2]. At present, several experimental techniques (such as filter binding assays, genetic analysis, chromatin immunoprecipitation on microarrays, and X-ray crystallography) have been used for identifying DNA-BPs. However, experimental approaches for identifying the DNA-BPs are costly and time consuming. Therefore, a reliable identification of DNA-BPs as well as DNA-binding sites with effective computational approach is an important research topic in the proteomics fields, which can play a vital role in proteome function annotation and discovery of potential therapeutics for genetic diseases and reliable diagnostics.

Computational prediction of proteins that interact with DNA is a difficult task, and state of the art methods have shown only limited success in this arena at present. Previously, there have been several machine-learning methods developed for prediction of DNA-BPs in the literature. Broadly, these methods can be divided into two categories: i) analysis from protein structure [3-6] and ii) prediction from amino acid sequence [7-12]. The accuracy of structure-based prediction methods is usually higher, but it can’t be used in high throughput annotation with the limited number of protein structures. Theoretically, the sequence of a protein contains all the necessary information to predict its function [13]. Until now, many methods for predicting protein function directly from amino acid sequences are useful tools in the study of uncharacterized protein families and in comparative genomics [14]. There are two major problems in the task of computational protein function prediction, which are the choice of the protein representation and the choice of the classification algorithm. To obtain good predictive model, various machine-learning algorithms such as support vector machine (SVM) [8,10,15-18], neural network [3,6,19], random forest [12,20], naïve Bayes classifiers [21,22], nearest neighbor [23] and ensemble classifiers [24,25] have been used to build classification models. Among these, the most widely used algorithm for prediction of DNA-BPs is SVM.

In context to the current study, SVM learns the features specific to the DNA-BPs and generates support vectors decisive for possible classification of any given sequence as DNA-BPs and achieved satisfactory results. The most important challenge for SVM-based prediction is to find a suitable way to fully describe the information implied in protein-DNA interactions [26]. There are several different protein features and feature extraction methods that can be used [8,27-29] and a comprehensive survey of these methods can be found in related research work [30,31]. However, the underlying principle of protein-DNA interactions is still largely unknown. It is desirable to explore the implications of those already identified features and newly undiscovered properties by machine learning approaches to further advance the prediction accuracy and understand the binding mechanism of DNA-BPs.

Thus, a systematic comparison of all protein features known to perform well is investigated in this article. We propose a novel method for predicting DNA-BPs using the SVM algorithm in conjunction with comprehensive feature analysis based on protein sequence. A recent work about mechanisms of protein folding research [32] has shown that the property factors of protein can be naturally clustered into two classes. One class is comprised of properties that favor sequentially localized interaction clusters; the other class is in favor of globally distributed interactions. Following the methodology introduced earlier in related protein function prediction work [29-31], we consider a feature vector (xi) to represent proteins which are derived from sequences broadly from three main levels: Global sequence descriptors, Nonlocal sequence descriptors and Local sequence descriptors. Feature vectors extracted from different sequence levels contain information about characteristics of the proteins at different scales which may be helpful in describing the information implied in protein-DNA interactions and improving the final model accuracy.

**Figure 1:**
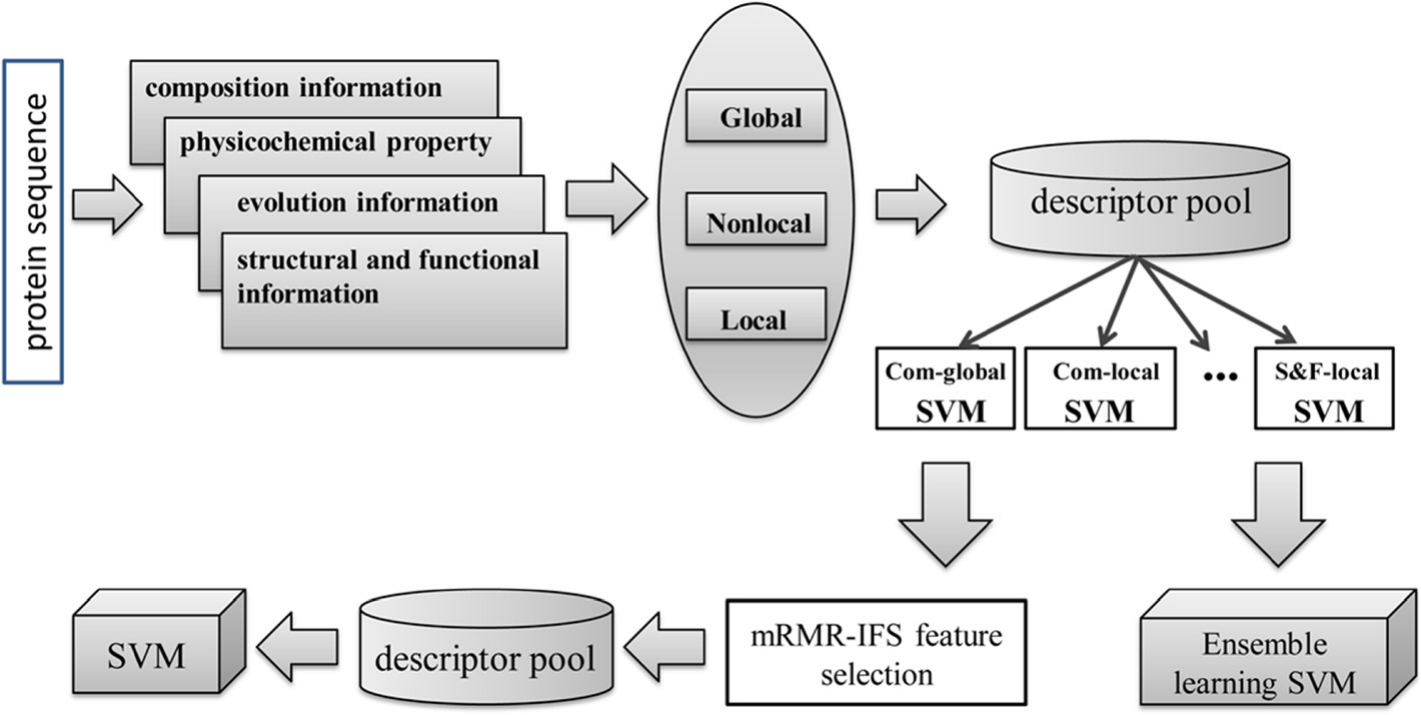
**The overall workflow of the present method.** Firstly, the input amino acid sequence is represented numerically by four kinds of features. Secondly, these feature values are transformed to feature descriptor matrices from three different levels. Thirdly, the first round of the evaluation is adopted based on the original descriptor pool and individual SVM models obtained. At last, mRMR-IFS feature selection method and ensemble learning approach are applied as the final evaluation of the optimal SVM model.

This paper consists of three main parts: Firstly, we investigate four different kinds of protein features which are composition information, structural and functional information, physicochemical properties and evolutionary information derived from reported literatures, public databases, and related prediction systems. The most informative and representative features are roughly derived from these four kinds of properties. Secondly, three different coding methods are adopted to represent different protein features selected above. These methods are called OCTD (Global method), auto covariance (AC) (Nonlocal method) and SAA (Local method). Lastly, the performance of different feature extraction strategies are extensively investigated by individual SVM classifiers, we collect and analyze those descriptors generated by means of good prediction behaviors. The mRMR-IFS feature selection approach and ensemble learning method are adopted to determine the ultimate prediction model. Our results show that accurate prediction of DNA-BPs can be obtained using a comprehensive analysis of global, nonlocal and local information of protein sequence together. The overall workflow of our method is shown in Figure 1.

## Methods

### Datasets

Four types of datasets are used here: i) DNAdset consists of partial sequences (binding regions or DNA binding domains) ii) DNAaset consists of full-length DNA-binding proteins. It’s reported that models trained on DNA domains or partial sequences are not suitable for predicting DNA binding proteins and vice versa, so separate methods are necessary for predicting DNA-binding domains and DNA-binding proteins [10]. iii) An independent test set called DNAiset used for testing and comparing. iiii) DNArset with non-equal number of positives and negatives used for evaluating our method in real life.

### DNAdset

The domain dataset also called DNAdset, consists of 231 DNA-BPs and 231 non-binding proteins with known structures which were obtained from a union of datasets used in previously related studies [8,9,16]. After clustering by CD-HIT [33] and careful inspection, these proteins have less than 40% sequence identity between each pair and without irregular amino acid characters such as “X” and “Z”. Thus the obtained DNAdset consists of 462 proteins, half of which are DNA-BPs and the other half are non-binding proteins. A complete list of all the PDB codes for DNAdset can be found in Additional file 1.

### DNAaset

To evaluate effectiveness of our methods by comparing with previously famous studies, we used the benchmark DNAaset from reported papers [10,17]. The dataset consists of 1153 DNA-binding proteins and 1153 non-binding proteins obtained from Swiss-Prot. No two protein sequences have similarity more than 25% and without irregular amino acid characters such as “X” and “Z”.

### DNAiset

In order to evaluate performance of our models on dataset not used for training or testing and compare with other reported methods, we obtained an independent dataset called DNAiset from newly determined DNA-binding protein structures from PDB by keyword searching (released on 2012-01-01 and later) and non-binding proteins used in a reported prediction method [12]. To reduce the redundancy and homology bias, CD-HIT [33] and PISCES [34] programs were used to ensure no two protein sequences have similarity more than 30% between DNAiset and two training sets (i.e. DNAdset and DNArset). Finally, the DNAiset has 80 DNA-binding protein chains selected from PDB and 192 non-binding proteins obtained from a newly developed web server named iDNA-Prot [12]. A complete list of all the PDB codes for DNAiset can be found in Additional file 1.

### DNArset

Equal number of positives and negatives is important for developing a powerful predictor for a protein system. It’s also important for evaluating the performance of the prediction model where one can simply calculate the accuracy. All the above datasets in our study have equal number of DNA-binding proteins and non-binding proteins. However, in a real-world situation, DNA-binding proteins are only a fraction of all proteins. It’s one of the (relatively) new problems called imbalanced dataset which has received an increasing attention since the workshop at AAAI 2000 [35]. This raises questions on whether models developed on equal numbers will be effective in real life. Will the method have a significantly poorer performance with more negatives in a test case? Thus, we created a more realistic dataset called DNArset to answer it. This dataset has 231 DNA-BPs used in DNAdset and 1500 non-binding proteins used by Kumar et al. as their “DNArset” [10].

### Support vector machine

Support vector machine (SVM) is a machine learning algorithm based on statistical learning theory presented by Vapnik (1998). It takes a set of feature vectors as the input, along with their output, which is used for training of model. Application of SVM in bioinformatics to various topics has been explored [16]. In this study, publicly available LIBSVM package version 3.11 [36] is used for the implementation of SVM and the RBF is taken as the kernel function, the tunable parameters are optimized based on grid search method to deliver high accuracy. All feature descriptors derived below were normalized in the range of [0, 1] by using formula (value-minimum)/ (maximum-minimum) before training SVM.

### Protein features

To develop a powerful function predictor for a protein system, one of the keys is to formulate the datasets with an effective mathematical expression that can truly reflect their intrinsic correlation with the attribute to be predicted [27]. To realize this, we assess four kinds of features including composition information, physicochemical property, evolutionary information and structural/functional property. The feature vector representations of these features are generated from three different levels, including Global sequence descriptors, Nonlocal descriptors and Local descriptors. Here, the composition information including overall amino acid composition (global descriptors), Dipeptide composition (nonlocal descriptors) and split amino acid composition (local descriptors). The other three kinds of properties are transformed by three different feature transformation methods which are introduced detailedly in Methods section.

### Overall amino acid composition (OAAC)

The conventional overall amino acid composition is defined as a 20-dimensional vector, which consists of the occurrence frequencies of 20 native amino acids. Given a protein *P*:

(1) $$\begin{array}{cc} p_{i}=\frac{n_{i}}{L} & \left(i=1,2\ldots ,20\right) \end{array}$$

Where *p*_*i*_ represents the occurrence frequency of the *i*-th native amino acid in the protein, *n*_*i*_ is the number of the *i*-th native amino acid in sequence, *L* is the length of the sequence in protein *P*.

It is reported that better performance could be obtained by calculating the square root of *p*_*i*_ instead [37]. The algorithm was inspired by the principle of superposition of state in quantum mechanics and proved good here. So we use *f*_*i*_ as overall amino acid composition (OAAC) information.

(2) $$\begin{array}{cc} f_{i}=\sqrt{p_{i}} & \left(i=1,2\ldots ,20\right) \end{array}$$

### Dipeptide composition (DPC)

**Figure 2:**
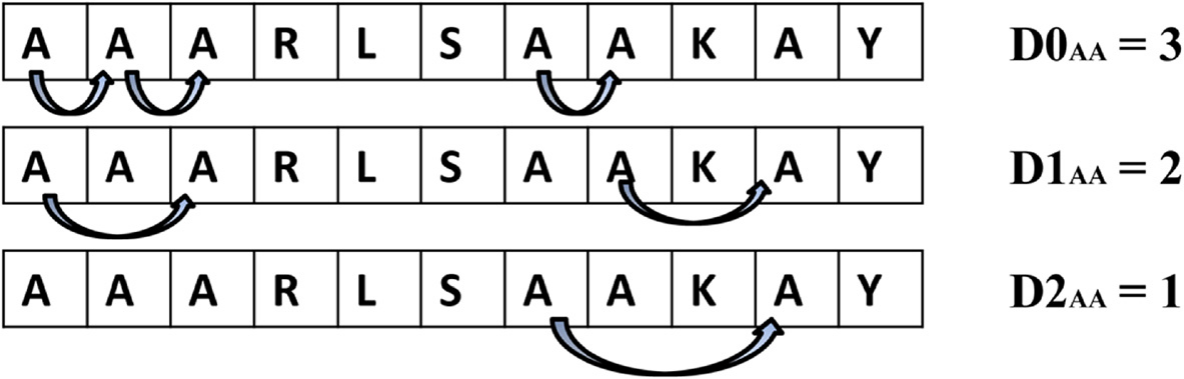
**The count of three kinds of Dipeptide composition *D0*, *D1*, *D2*.**

Dipeptide composition (DPC) comprises of two consecutive residues which gives a fixed pattern length of 400. This widely used sequence representation encapsulates information about the fraction of amino acids as well as their local order [38]. In this paper, firstly, three kinds of Dipeptide composition *DP*_*0*_, *DP*_*1*_, *DP*_*2*_ are calculated by counting all pairs of amino acid condition with 0, 1 and 2 skips respectively [22] as shown in Figure 2. Each kind of Dipeptide composition gives 400 descriptors, defined as:

(3) $$\begin{array}{cc} f_{s}\left(i,j\right)=\frac{D_{s}\left(i,j\right)}{N-1} & \left(\begin{array}{cc} i,j=1,2,3,\ldots ,20 & s=0,1,2 \end{array}\right) \end{array}$$

Where *D*_*s*_(*i*, *j*) is the number of Dipeptide represented by amino acid type *i* and *j* with *s* skips, *f*_*s*_(*i*, *j*) represents the occurrence frequency. *N* is the length of the sequence.

Then, we concatenate the vector elements of *DP*_*0*_, *DP*_*1*_ and *DP*_*2*_ together and the mRMR method [39] is adopted to select the first 400 descriptors from the total of 1200 dimensions as DPC used in this paper.

### Split amino acid composition (SAAC)

Split Amino Acid Composition (SAAC) was introduced where the protein sequence is divided into three parts: N terminal, C terminal and a region between them and composition of each part is calculated separately according to equations (1) and (2). Many previous literatures adopted SAAC for protein function prediction and achieved good results [40,41]. In our SAAC method, the detailed strategy of splitting the protein sequence is illustrated in Split amino acid (SAA) Transformation section.

### Physicochemical properties

Physicochemical properties of amino acids have been successfully employed in many sequence based function predictions with characteristics of well defined and high interpretability [17,40,42]. AAIndex [43] is a well known database of amino acid biochemical and physicochemical properties. Recently, a systematic approach (named Auto-IDPCPs) has been conducted to identify informative physicochemical and biochemical properties in the AAIndex database to design SVM-based classifiers for predicting and analyzing DNA-BPs [17]. We use the selected 28 representative numerical index scores in their paper to encode each amino acid in this study, as shown in Table 1. A complete protein sequence is then represented by a set of 28×*L* numerical strings, each of which records the course of one property value along the *L*-residue sequence.

**Table 1:** List of the AAIndex indices used in this paper

**Feature ID**	**AAIndex ID**	**Feature description**
39	CHOP780202	Normalized frequency of beta-sheet (Chou-Fasman, 1978b)
56	CIDH920103	Normalized hydrophobicity scales for alpha+beta-proteins (Cid et al., 1992)
58	CIDH920105	Normalized average hydrophobicity scales (Cid et al., 1992)
86	FAUJ880109	Number of hydrogen bond donors (Fauchere et al., 1988)
88	FAUJ880111	Positive charge (Fauchere et al., 1988)
95	FINA910104	Helix termination parameter at posision j+1 (Finkelstein et al., 1991)
100	GEIM800104	Alpha-helix indices for alpha/beta-proteins (Geisow-Roberts, 1980)
102	GEIM800106	Beta-strand indices for beta-proteins (Geisow-Roberts, 1980)
139	KANM800102	Average relative probability of beta-sheet (Kanehisa-Tsong, 1980)
146	KLEP840101	Net charge (Klein et al., 1984)
147	KRIW710101	Side chain interaction parameter (Krigbaum-Rubin, 1971)
167	LIFS790101	Conformational preference for all beta-strands (Lifson-Sander, 1979)
178	MEEJ800101	Retention coefficient in HPLC, pH7.4 (Meek, 1980)
214	OOBM770102	Short and medium range non-bonded energy per atom (Oobatake-Ooi, 1977)
229	PALJ810107	Normalized frequency of alpha-helix in all-alpha class (Palau et al., 1981)
280	QIAN880123	Weights for beta-sheet at the window position of 3 (Qian-Sejnowski, 1988)
299	RACS770103	Side chain orientational preference (Rackovsky-Scheraga, 1977)
321	RADA880108	Mean polarity (Radzicka-Wolfenden, 1988)
356	ROSM880102	Side chain hydropathy, corrected for solvation (Roseman, 1988)
365	SWER830101	Optimal matching hydrophobicity (Sweet-Eisenberg, 1983)
399	ZIMJ680102	Bulkiness (Zimmerman et al., 1968)
401	ZIMJ680104	Isoelectric point (Zimmerman et al., 1968)
422	AURR980120	Normalized positional residue frequency at helix termini C4’ (Aurora-Rose, 1998)
431	MUNV940103	Free energy in beta-strand conformation (Munoz-Serrano, 1994)
449	NADH010104	Hydropathy scale based on self-information values in the two-state model (20% accessibility) (Naderi-Manesh et al., 2001)
451	NADH010106	Hydropathy scale based on self-information values in the two-state model (36% accessibility) (Naderi-Manesh et al., 2001)
512	GUYH850105	Apparent partition energies calculated from Chothia index (Guy, 1985)
528	MIYS990104	Optimized relative partition energies - method C (Miyazawa-Jernigan, 1999)

### PSSM profiles

To use the evolution information, the position-specific scoring matrix (PSSM) [44] profiles are adopted, which have been widely used in protein function prediction and other bioinformatics problems with notable improvement of performance [10,45]. Here, the PSSM profiles are generated by using the PSI-Blast program [44] to search the non-redundant (NR) database (released on 14 May 2011) through three iterations with 0.001 as the E-value cutoff for multiple sequence alignment. The final PSSM scoring matrix has 20×*L* elements (excluding dummy residue X), where *L* is the length of protein.

### Secondary structure composition

Secondary structure is an important structural feature of protein that can significantly improve the function prediction performance [46,47]. In this study, secondary structure calculation is carried out by PSIPRED v3.0 [48], which is one of the state-of-the-art protein secondary structure prediction methods with an accuracy of up to 80%. PSIPRED predicts secondary structure for each residue in a protein and provides a confidence score for three types of secondary structures: helices, β-sheets and coil regions, thus we get 3×*L* feature values where *L* is the length of protein.

### Disorder feature score

Over the past decade, there has been a growing acknowledgement that a large proportion of proteins within most proteomes contain disordered regions. Protein with disordered regions can play important functional roles. Its flexibility is advantageous to proteins that recognize multiple target molecules including biomacromolecules like DNA with high specificity and low affinity [49-51]. The IUPred [52] method is used to score the disorder status of each amino acid which recognizes intrinsically disordered protein regions from amino acid sequences by estimating their total pairwise interresidue interaction energy. The prediction type option for IUPred is set as long and we get *L* feature values where *L* is the length of protein.

### Feature transformation method

Three different methods are used here to transform pre-selected protein features into uniform length descriptors to capture various types of information implied in proteins. The following part describes in detail the methodology for each of these different transformation methods.

### Overall composition-transition-distribution (OCTD)

The original CTD method was first introduced by Dubchak et al. [53] as a global description of protein sequence for predicting protein folding class. Recently, CTD has been adopted by more and more leading investigators for protein function and structure studies [54,55] . Composition (C) is the number of amino acids of a particular property divided by the total number of amino acids. Transition (T) characterizes the percent frequency with which amino acids of a particular property is followed by amino acids of a different property. Distribution (D) measures the chain length within which the first, 25, 50, 75 and 100% of the amino acids of a particular property is located respectively. Here, we develop a new variant method of CTD named Overall Composition-Transition-Distribution (OCTD). Initially, we represent the sequence numerically for a particular feature. Then, we normalize the feature values in the range of [0, 1] using formula (value-minimum)/ (maximum-minimum), amino acids were grouped into two classes according to its feature values of threshold 0.5. Finally, the CTD method is adopted to represent the amino acid properties distribution pattern of a specific property along the protein sequence.

### Autocross-covariance (ACC) Transformation

The Autocross-covariance (ACC) method is a simplified nonlocal statistical tool for analyzing sequences of vectors which developed by Wold et al. [56]. Recently, ACC has been adopted by many protein function prediction studies [57,58] including DNA-BPs [8]. ACC method results in two kinds of variables, AC between the same kind of descriptors, and cross covariance (CC) between two different descriptors. In this study, only AC variables are used in order to avoid generating too large number of descriptors and based on the observations from previously related studies [58]. AC variables describe the average interactions between two residues, a certain *lg* apart throughout the whole sequence. Here, *lg* is the distance between one residue and its neighbor, a certain number of residues away. The AC variables are calculated according to Equation (4) below:

(4) $$AC_{\left(i,\mathrm{lg}\right)}=\sum_{j=1}^{L=\mathrm{lg}}\left(S_{i,j}-\bar{S_{i}}\right)\left(S_{i,j+\mathrm{lg}}-\bar{S_{i}}\right)/\left(L-\mathrm{lg}\right)$$

Where *i* is one of the properties, *j* is the position in the sequence, *L* is the length of the protein sequence *S*_*i,j*_ is the feature value of *i* at position *j*, 

$\bar{S_{i}}$ is the average score for amino acid *i* along the whole sequence:

(5) $$\bar{S_{i}}=\sum_{j=1}^{L}S_{i,j}/L$$

Thus, the number of AC variables can be calculated as *P*×*LG*, where *P* is the number of feature value, *LG* is the maximum of *lg* (*lg*=1,2,…,*LG*).

### Split amino acid (SAA) Transformation

There have been several ways to calculate protein local features, the proposed method split amino acid composition (SAAC) where composition of N-terminal, middle and C-terminal of protein is computed separately [40,41]. The PNPRD method which divided the protein sequence into 10 local regions based on positively and negatively charged residues [59] is some kind of variation from CTD method. There are also more concise method by splitting each protein into 10 local regions of varying length and CTD method was used to exact descriptors from local regions [60,61].

Here, we adopt the powerful split amino acid (SAA) method to represent local composition of different protein features selected before. Firstly, each sequence is split into three parts: N-terminal, middle and C-terminal. The N-terminal part is further divided into four regions to consider ambiguity in the length and position of signal sequences. Then, the mean of different features corresponding to the six divided local regions are obtained to generate a fixed number of local descriptors.

**Figure 3:**
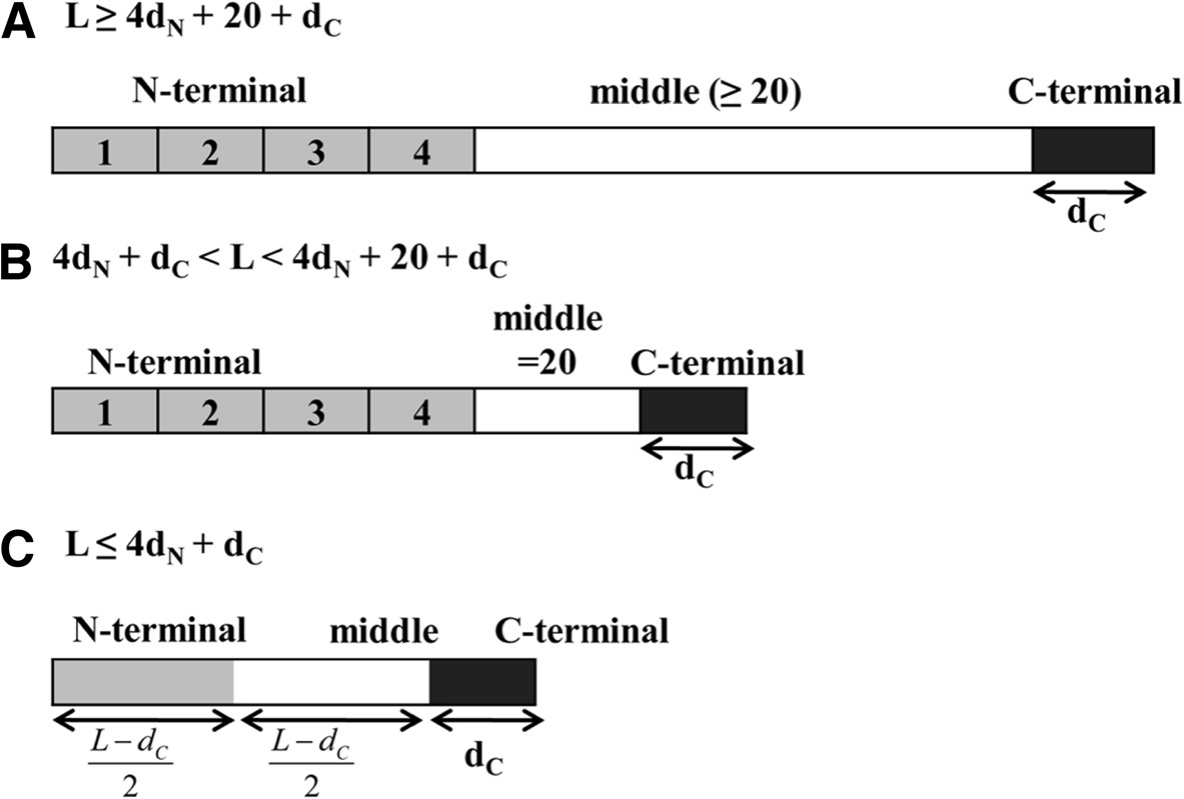
**Definitions of the N-terminal, middle, and C-terminal parts depending on sequence length *L *for SAA method.**

We define the N-terminal, middle and C-terminal parts depending on sequence length *L*. The N-terminal part is further divided into four regions with length *d*_N_ which is set to 25. It’s also assumed that the middle part *d*_M_ has at least 20 residues equal to the number of distinct amino acids. The length of the C-terminal part *d*_C_ is set to 10 (Figure 3A). For short sequences, we use two more definitions. If *L* is > 4*d*_N_+*d*_C_ and < 4*d*_N_+20+*d*_C_, the middle part is regarded as 20 residues from the start of the C-terminal part toward the N-terminal part (Figure 3B). In the case that *L* is ≤ 4*d*_N_+*d*_C_, we assumed that the lengths of the N-terminal and middle parts are the same which are equal to (*L* - *d*_C_)/2 and the N-terminal part is not divided at all (Figure 3C).

### Features selection

**Maximum Relevance, Minimum Redundancy (mRMR)** method was first proposed by Peng for processing microarray data [39]. Max-Relevance means that candidate feature to be selected preferentially has the maximal correlation with the target variable, while Min-Redundancy requires that candidate feature to be selected preferentially has minimal redundancy with the features already selected. Both Relevance and Redundancy are measured by the mutual information (MI) defined as:

(6) $$I\left(x,y\right)-\iint p\left(x,y\right)\log\frac{p\left(x,y\right)}{p\left(x\right)p\left(y\right)}\mathrm{dxdy}$$

Where *x* and *y* are two random variables, *p*(*x*, *y*) is the joint probabilistic density, *p*(*x*) and *p*(*y*) are the marginal probabilistic densities respectively. The mathematical description of the algorithm was detailedly presented in the Peng’s previous study [39]. To calculate MI, the joint probabilistic density and the marginal probabilistic densities of the two vectors were used. A parameter *t* is introduced here to deal with these variables. Suppose mean to be the average value of one feature in all samples, and std to be the standard deviation, the feature of each sample would be classified into one of the three groups according to the boundaries: mean ± (*t*×std). Here, *t* was assigned to be 1.

After mRMR procedure, mRMR feature pool ***S*** (shown as eq(7)) containing all features in an ordered way is obtained. Now we know the advantages of the features, but we do not know how many features and which features we should choose. Incremental Feature Selection (IFS) step was utilized to determine the optimal number of features and the optimal features based on mRMR method.

(7) $$S=\left\{f_{0},f_{1},\ldots ,f_{h},\ldots ,f_{N-1}\right\}$$

**Incremental Feature Selection (IFS)** According to mRMR result, we can construct the *N* feature sets from ordered feature set ***S*** (eq(7)) as follows:

(8) $$\begin{array}{cc} S_{i}=\left\{f_{0},f_{1},\ldots ,f_{1}\right\} & \left(0\leq \;i\leq \;N-i\right) \end{array}$$

Where *f*_*i*_ is the *i*-th sorted feature in the feature list.

For each feature subset, we use SVM to construct predictor which is evaluated by five-fold cross-validation. As a result, we get a curve named IFS curve, with MCC value as its *y*-axis and index *i* of *S*_*i*_ as its *x*-axis. When the overall IFS curve reaches at the peak, meanwhile, the corresponding predictor is chosen as the ultimate prediction model.

### Ensemble learning method

The idea of ensemble learning methodology is to build a predictive model by integrating multiple models, treating them as a committee of decision makers. As a growing body of studies indicates that every single learning strategy has its own shortcomings and none of them could consistently perform well over all datasets. To overcome this problem, ensemble methods have been suggested as the promising measures [62,63]. In general, an ensemble consists of a set of models and a method to combine them. We have twelve different SVM models after the first round of evaluation for exploring the performance of SVM-based modules constructed by different types of features (see Results section for details). Two popular model combination strategies: majority voting and stacking are adopted here.

In majority voting scheme, a classification of an unlabeled instance is performed according to the predicted class that obtains the highest number of votes. That is, we have twelve different classifiers in this work, if a majority of the twelve modules predict a protein as binding, then the prediction result of this protein is taken as binding. When equal number occurred, we found most proteins in this situation are non-binding so the threshold is assigned to six for binding prediction in majority voting scheme.

Stacking is a technique for achieving the highest generalization accuracy [25,63]. Different from the majority voting scheme, stacking will learning twice. The basic idea is to learn a function that combines the predictions from the individual classifiers. Instead of using the original input attributes, it uses the predicted probabilities for every class label from the base-level classifiers as the input attributes of the target for the second-level machine learning. In our work, we use the decision values from the twelve SVM modules as the input feature vectors for final meta SVM-predictor in stacking scheme.

### Performance evaluation

Six parameters are employed to indicate the performance of our method, including overall accuracy (Acc), area under the receiver operating characteristic curve (AUC), F-score, sensitivity (Sen), specificity (Sp) and Matthews’s correlation coefficient (MCC). Details of these indices are listed in Table 2. The five-fold cross validation method is used to evaluate model which can minimize the overfitting of the prediction model, the whole dataset is randomly separated into five parts. Each time, one part is retained for testing and all others form the training dataset. This process is repeated five times to test each subset. The evaluation parameters above are calculated as the average from the 5-fold cross validation.

**Table 2:** Indices used to evaluate the prediction method

**Index**	**Definition and formula**
Acc	(*TP* + *TN*)/(*TP* + *TN* + *FP* + *FN*)
AUC	area under the receiving operating characteristic curve
F-score	2 · *TP*/(2*TP* + *FP* + *FN*)
Sen	*TP*/(*TP* + *FN*)
Sp	*TN*/(*TN* + *FP*)
MCC	$\frac{\mathrm{TP}\cdot \mathrm{TN}-\mathrm{FN}\cdot \mathrm{FP}}{\sqrt{\left(\mathrm{TP}+\mathrm{FN}\right)\cdot \left(\mathrm{TP}+\mathrm{FP}\right)\cdot \left(\mathrm{TN}+\mathrm{FN}\right)\cdot \left(\mathrm{TP}+\mathrm{FP}\right)}}$

TP (true positive); TN (true negative); FP (false positive); FN (false negative);

Acc, overall accuracy; AUC, area under ROC; Sen, sensitivity; Sp, specificity; F-score, 2×precision×sensitivity/(precision+sensitivity); MCC, Matthews’s correlation coefficient.

## Results

### AC results

**Figure 4:**
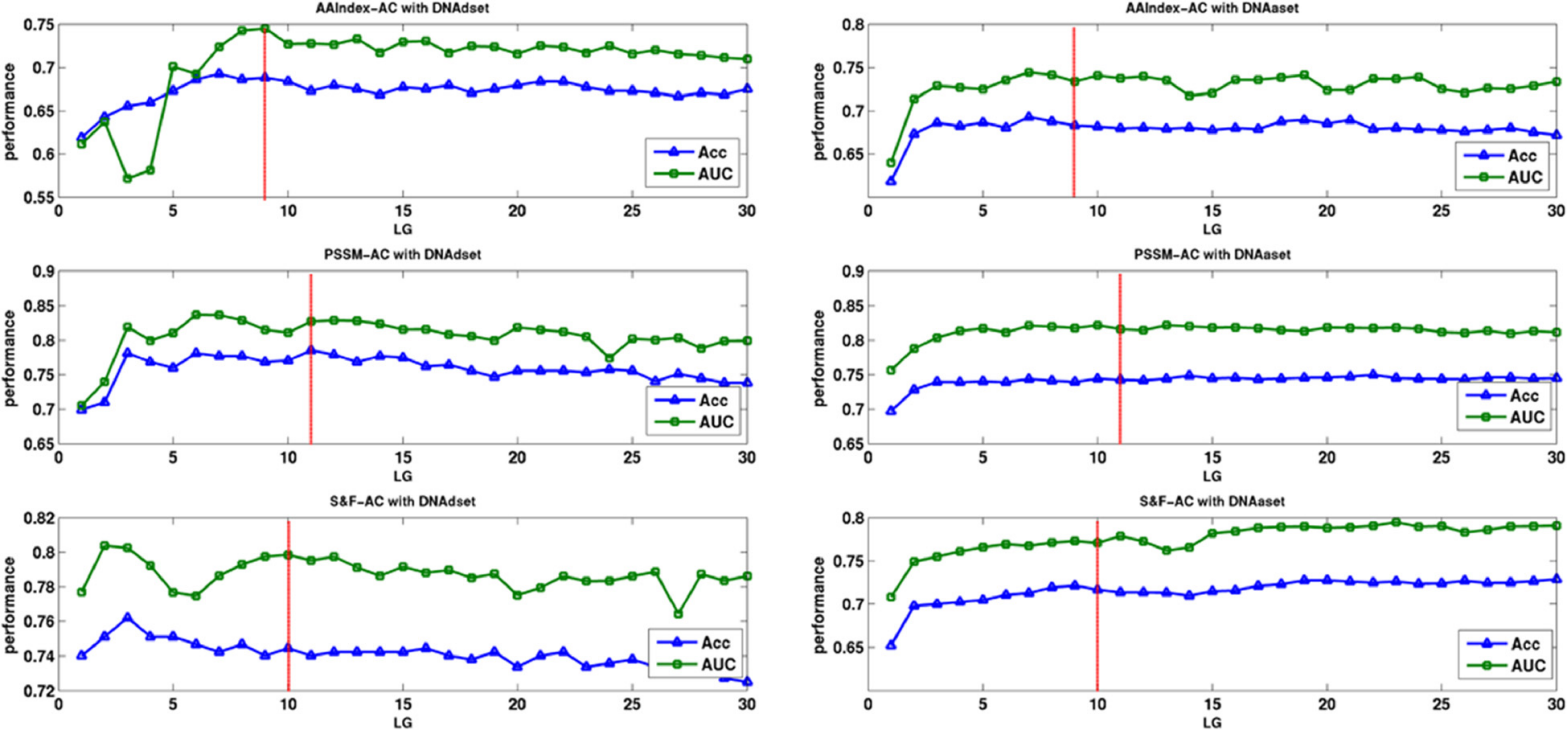
**The performance of different AC features with various *LG *values over DNAdset and DNAaset.**

To evaluate the AC transformation method, we first check the impact of parameter *LG* to achieve the best characterization of the protein sequence. Here, we use both Acc and AUC parameters as optimization objectives to determine the optimal values of *LG* for each feature transformation. The performance of different AC transformed features for different values of *LG* on the DNAdset and DNAaset is shown in Figure 4. To make a uniform representation, the optimal values of *LG* for AAindex-AC, PSSM-AC and S&F-AC (S&F means the Structural and Functional information which include Secondary Structure Composition and Disorder Feature Score) are set to 9, 11 and 10 respectively in both DNAdset and DNAaset.

### Individual SVM-modules results

The detailed procedure of our method is illustrated in Figure 1. The details about the selected properties of protein sequence and how the feature descriptor matrices were compiled are outlined in Methods section. During the first round of the evaluation, we explore the performance of SVM-based modules constructed by different features transformed from various levels. They are termed OAAC, DPC, SAAC, AAIndex-OCTD, AAIndex-AC, AAIndex-SAA, PSSM-OCTD, PSSM-AC, PSSM-SAA, S&F-OCTD, S&F-AC and S&F-SAA respectively. The prediction results of individual SVM-modules using 5-fold cross-validation over the DNAdset and DNAaset are tabulated in Table 3. As shown in Table 3, most of these descriptors are well performed in discriminating the DNA-BPs from non-DNA-BPs as expected. The reason is that those carefully selected informative features are well performed ones in previous studies or some inherent properties related to protein-DNA interactions. Moreover, it demonstrates the usefulness of feature transformation methods used here. Sequence composition information is the basic characters of protein sequences and modules based on the three kinds of composition descriptors are well performed as expected. For predicting DNAaset, PSSM profiles transformed by SAA method are more powerful than commonly used PSSM-400 [10] which has an accuracy of 74.22% and MCC of 0.49. The performances of PSSM-OCTD and PSSM-AC are equal to PSSM-400 method. There is no much difference among three transformation methods for S&F feature, but moderate performances in both DNAdset and DNAaset are noticeable not only because of the difference between the two datasets but also because of the limited accuracy of PSIPRED and IUPred which is just around 80%. So we believe that protein secondary structure and disorder patterns are closely connected with the process of protein-DNA interactions and some related papers have discussed on it [49,64]. In the future, we believe the prediction power of combined S&F features will significantly improve when more accurate prediction methods of secondary structure and disorder patterns are developed. For AAIndex features transformed by OCTD method, the Acc parameter is 0.743, a little less than the Auto-IDPCPs method which used the same 28 AAIndex [17] with an accuracy of 0.755. The poor performance of the nonlocal descriptors AAIndex-AC is somewhat unexpected as we speculated that the coding method should be able to capture some information of amino acid physicochemical and biochemical properties related to the nonlocal nature. However, previous research has demonstrated the existence of only two types of physicochemical and biochemical properties which are locally and globally distributed [32]. So, we are continuing to investigate the implications of these observations.

**Table 3:** The performance of different kinds of feature descriptors

**Descriptor**	**DNAdset**						**DNAaset**					
	**Acc**	**AUC**	**F-score**	**Sen**	**Sp**	**MCC**	**Acc**	**AUC**	**F-score**	**Sen**	**Sp**	**MCC**
OAAC	0.872	0.941	0.856	0.865	0.852	0.716	0.726	0.794	0.742	0.799	0.650	0.451
DPC	0.872	0.925	0.838	0.865	0.809	0.672	0.717	0.784	0.725	0.753	0.682	0.436
SAAC	0.846	0.904	0.826	0.842	0.813	0.651	0.697	0.740	0.701	0.743	0.624	0.369
AAIndex-OCTD	0.845	0.905	0.824	0.828	0.825	0.651	0.743	0.782	0.729	0.766	0.664	0.452
AAindex-AC	0.688	0.745	0.707	0.729	0.680	0.410	0.683	0.734	0.705	0.785	0.559	0.353
AAIndex-SAA	0.870	0.915	0.840	0.869	0.811	0.678	0.708	0.747	0.732	0.808	0.601	0.417
PSSM-OCTD	0.729	0.776	0.721	0.728	0.724	0.452	0.741	0.811	0.742	0.745	0.738	0.483
PSSM-AC	0.786	0.827	0.762	0.771	0.752	0.523	0.742	0.816	0.734	0.725	0.751	0.477
PSSM-SAA	0.872	0.932	0.872	0.903	0.839	0.741	0.761	0.840	0.773	0.797	0.737	0.535
S&F-OCTD	0.723	0.801	0.737	0.714	0.779	0.493	0.719	0.770	0.711	0.726	0.694	0.411
S&F-AC	0.745	0.799	0.729	0.756	0.690	0.446	0.717	0.771	0.701	0.690	0.723	0.413
S&F-SAA	0.712	0.734	0.649	0.627	0.736	0.371	0.711	0.768	0.703	0.710	0.692	0.402

After investigating individual coding scheme, it’s confirmed that all twelve kinds of descriptors are reasonable for discriminating DNA-BPs. We intend to combine the well performed descriptors above-mentioned which mean a comprehensive presentation of protein functional features related to DNA binding. Thus, two different strategies are proposed here for achieving this goal which are ensemble learning and feature selection.

To achieve the reduction of noise and redundancy to improve the classification accuracy and the combination of more interpretable features that can help identify DNA-BPs, the proposed mRMR-IFS feature selection framework is adopted.

### MRMR results

The mRMR program in this study is downloaded from http://penglab.janelia.org/proj/mRMR/. Using the mRMR program, we obtain the ranked mRMR list of the first 1000 features from the original 2040 descriptors for DNAdset and DNAaset separately. Within the list, a smaller index of a feature indicates that it is deemed as a more important feature in the prediction. The mRMR list is retained and will be used in the IFS procedure for feature selection and further analysis.

### IFS results

**Figure 5:**
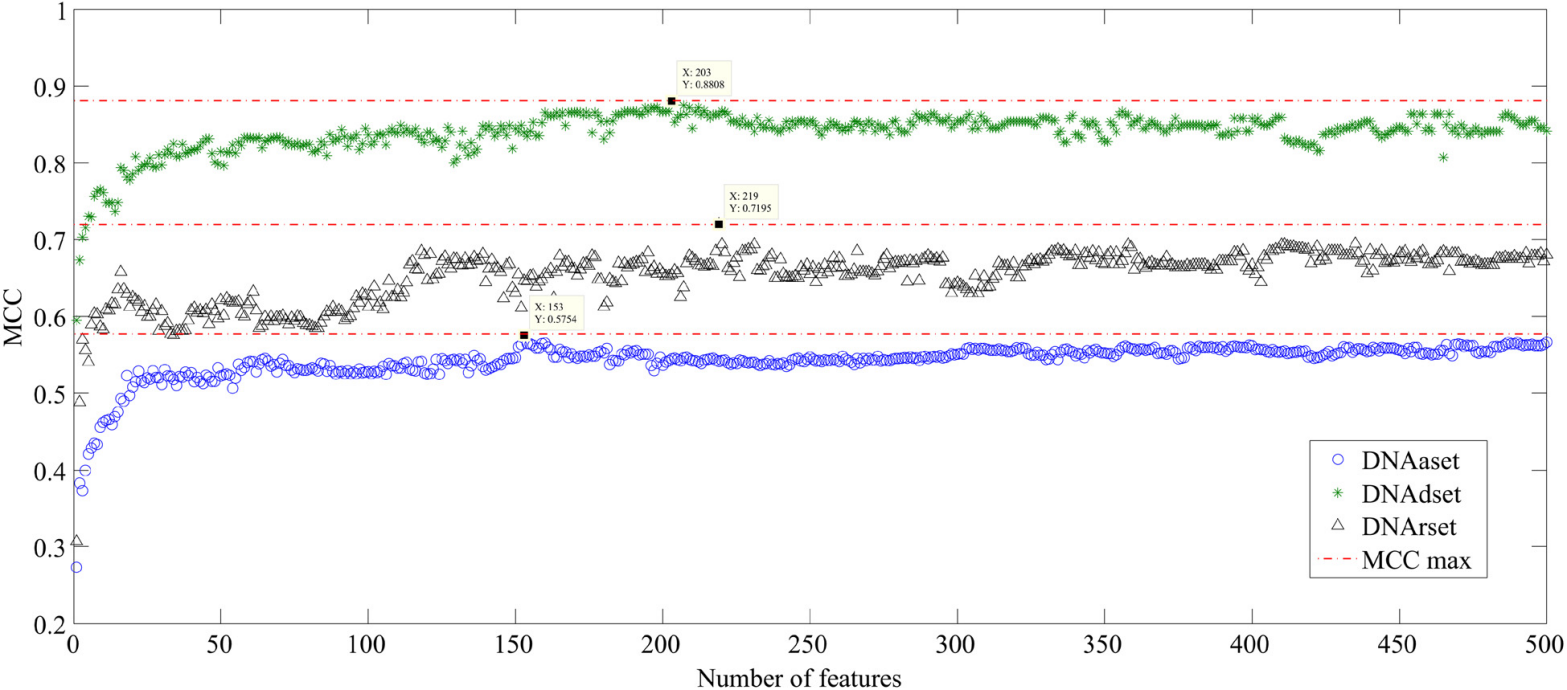
The IFS curves of DNAdset, DNArset and DNAaset.

On the basis of the outputs of mRMR, we built individual predictors by adding features recursively from the top of the mRMR list to the bottom. As shown in Figure 5, the plotted IFS curve and the detailed IFS results can be found in Additional file 2 and Additional file 3. As we can see, the maximum MCC is 0.881 (accuracy is 0.940) with 203 features and 0.575 (accuracy is 0.789) with 153 features for DNAdset and DNAaset respectively. So, these selected features are considered as the optimal feature set used in our final prediction model.

### Ensemble learning results

Based on the results of individual SVM-modules, ensemble learning method attempts to combine different models into a consensus classifier by majority voting and stacking method. The performance of ensemble learning with different combining method is listed in Table 4. As shown in Tables 3 and 4, the ensemble learning results are much better than those individual meta predictors including the excellent component classifier such as PSSM-SAA and DPC. Among the two kinds of combining method, stacking (with accuracy of 0.907 and 0.811 for DNAdset and DNAaset) outperforms majority voting (with accuracy of 0.898 and 0.789 respectively) which may benefit from the discrimination power of SVM and the idea of divide-and-conquer.

**Table 4:** The performance of feature-selection method and ensemble learning

**Method**	**DNAdset**						**DNAaset**					
	**Acc**	**AUC**	**F-score**	**Sen**	**Sp**	**MCC**	**Acc**	**AUC**	**F-score**	**Sen**	**Sp**	**MCC**
mRMR-IFS	0.940	0.973	0.940	0.964	0.917	0.881	0.789	0.864	0.793	0.819	0.766	0.575
Ensemble-voting	0.898	N/A	0.900	0.905	0.892	0.797	0.789	N/A	0.792	0.801	0.778	0.579
Ensemble-stacking	0.907	0.965	0.910	0.935	0.878	0.819	0.811	0.885	0.808	0.814	0.799	0.614

N/A – not available.

### Performance on independent dataset

In this study, we further evaluated the performance of our optimal SVM models (trained on DNAdset) on an independent dataset called DNAiset, which consists of 80 DNA-BPs and 192 non-binding proteins. The feature-selection-based model correctly predicted 72 and 170 out of 80 DNA-BPs and 192 non-binding proteins respectively, while the ensemble-based model correctly predicted 68 and 166 out of 80 DNA-BPs and 192 non-binding proteins respectively. This demonstrates that our SVM models perform equally well on the independent dataset.

### Performance on realistic dataset

In a real-world situation, the number of non-binding proteins is significantly higher than DNA-BPs. Thus, it is important to build and evaluate SVM models on more realistic data rather than equal number of DNA-BPs and non-binding proteins. Hence, we obtained a realistic dataset (DNArset), which has 231 DNA-BPs and 1500 non-binding proteins. Firstly, we developed SVM model using feature selection based method on DNArset and achieved the maximum MCC of 0.720 with an accuracy of 94.16%. Then we also developed ensemble learning models and the ensemble stacking method which achieved the maximum MCC of 0.729 with an accuracy of 94.28%, while the majority voting method has a significant poorer performance with more negative proteins which demonstrates the instability of it. The detailed five fold cross validation results and mRMR-IFS results are shown in Additional File 4. Lastly, we applied models trained by DNArset with feature selection method on DNAiset which can correctly predict 60 out of 80 positives and 172 out of 192 negatives (see Additional file 4). The results further confirmed the prediction effectiveness of our method.

### Comparison with existing methods

It is important to compare the performance of our protocol with existing methods in order to evaluate its effectiveness. The performance of feature-selection based models and ensemble learning models for DNA-binding prediction by fivefold cross-validation test is summarized in Table 4. The feature-selection based protocol has an accuracy of 0.940 and 0.789 for DNAdset and DNAaset respectively, accordingly ensemble learning protocol returns a little lower accuracy of 0.907 for DNAdset but a higher accuracy of 0.811 for DNAaset. For DNAaset, the performances of previously reported studies developed from it are [10], with accuracy of 0.742, MCC of 0.49, Sen of 0.735 and Sp of 0.749 and [17], with accuracy of 0.755, MCC of 0.51, Sen of 0.820 and Sp of 0.690. As shown in Table 4, the best performance in our protocol with an accuracy of 0.811, MCC of 0.614, Sen of 0.814 and Sp of 0.799 is much better.

As mentioned above, the DNAdset is a union of three datasets used in previously related studies, it has difficulty in direct comparison. So we adopted the newly developed independent test set called DNAiset to compare our method with several famous web-based servers. Our method correctly predicted 72 out of 80 positives and 170 out of 192 negatives. DNAbinder developed by Kumar et al. [10] can correctly predict 69 and 126 out of 80 DNA-BPs and 192 non-binding proteins respectively for DNAiset (the SVM model trained on main dataset was used). DNABIND developed by Szilagyi et al. [65] which correctly predicts 61 and 168 out of 80 DNA-BPs and 192 non-binding proteins respectively for DNAiset. The iDNA-Prot [12], adopted to select the DNAiset in this study, can correctly predict 67 out of 80 DNA-BPs and 171 out of 192 negatives. It should be noticed that the 192 non-binding proteins in DNAiset were used as training set in this method. Detailed comparison results of different methods can be found in Table 5. It demonstrates that both of our mRMR-IFS feature-selection method and ensemble learning strategy return satisfactory results and outperform some previous studies.

**Table 5:** Comparison of the predicted results by our method and some web-servers on DNAiset

**Method**	**Acc**	**F-score**	**Sen**	**Sp**	**MCC**
**DNA-Binder**	0.717	0.642	0.863	0.656	0.473
**DNABIND**	0.842	0.739	0.763	0.875	0.627
**iDNA-Prot**	0.875	0.798	0.837	0.891	0.709
**Our method**	0.890	0.828	0.900	0.886	0.753

## Discussion

**Figure 6:**
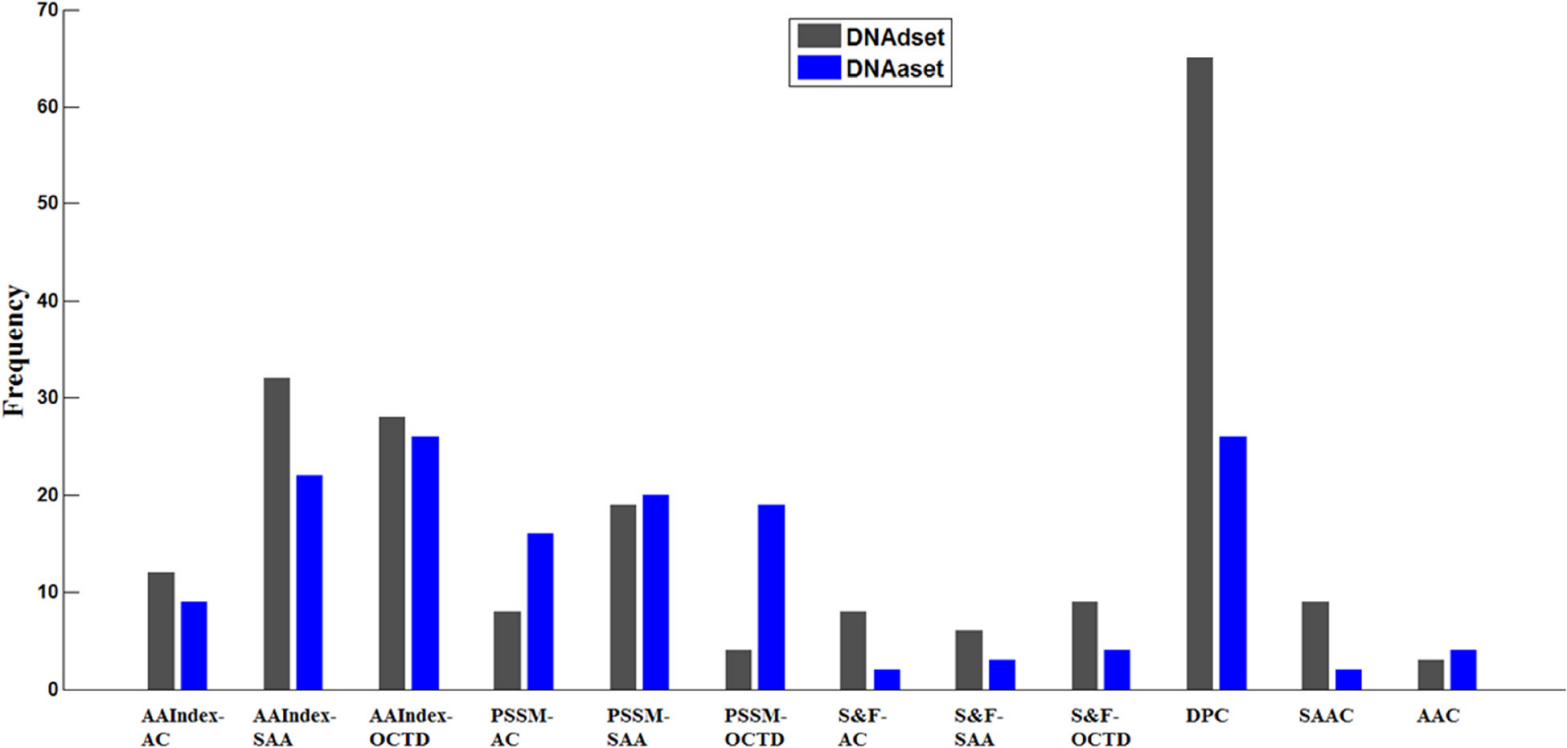
Distribution of the number of each type of features (a total 12 types) in the optimal feature set.

As described in the Methods section, there were four kinds of features transformed by three different methods which produced totally twelve types of feature vectors in this study. For both datasets, the original feature space of 2040 dimensions is reduced to 203 and 153 dimensions separately after mRMR-IFS feature selection process. The distribution of the number of each type of features in the optimal feature set is investigated and shown in Figure 6. As we can see from Figure 6, the proportion of each type of feature is similar between DNAdset (with 203 optimal features) and DNAaset (with 153 optimal features) except PSSM-OCTD features. Features of DPC, AAIndex features transformed by SAA and OCTD method and PSSM scores transformed by SAA method are over half of the optimal features and DPC is the highest. After further inspection of DPC in the optimal features we find positively charged lysine or arginine or aromatic phenylalanine most frequently appeared in these optimal two-tuples. These three kinds of amino acids play an important role in protein-DNA interactions as described in previous studies [66,67]. The other eight kinds of features like AAIndex-AC, PSSM-AC, etc. are also involved in the optimal features. This phenomenon suggests that all twelve kinds of features contribute to the prediction of protein-DNA interactions and that DPC features may play an irreplaceable role for DNA-BPs prediction. Further analysis in DNAdset reveals that physicochemical property and evolutionary information are better represented as local descriptors (by SAA transformation method), while DPC as nonlocal features of composition information is more befitting than others, and the structural/functional property tend to be globally and nonlocally represented (by OCTD and AC method). The same situation can be found in DNAaset, in addition, physicochemical property and evolutionary information transformed by OCTD are equal to SAA, and this isn’t hard to understand because DNAdset consists of partial sequences (binding regions or DNA binding domains) while DNAaset consists of full length. Thus global features for both AAIndex and PSSM transformed by OCTD in DNAdset are not really global like DNAaset. The results confirm that different properties of amino acid are preferentially distributed in various scales [32]. On the whole, no single type of feature could undertake the task of DNA-BPs prediction accurately. The most important challenge is to find a suitable way to fully describe the information of protein which is just what we are trying to do.

## Conclusions

In this work, we investigate the idea of ensemble of informative features from different levels for predicting DNA-BPs which is motivated by a recently research result that amino acid physical properties can fall into distinct levels [32]. The overall protocol is aimed at representing the four important kinds of properties of protein appropriately by different transformation methods and seeking the optimal feature descriptors for presentation of DNA-BPs. The performances of individual modules indicate the usefulness of features from various levels and their dissimilarity. Based on the obtained different kinds of feature descriptors, we take two strategies for the construction of the final prediction models which are mRMR-IFS feature selection protocol and ensemble learning approach. Encouragingly, we get good performance of Acc of 0.940 for DNAdset with the mRMR-IFS method and Acc of 0.811 for DNAaset with ensemble learning approach, and the performance on independent test set is also good.

Our experiments indicate that it may be helpful to develop a successful machine method to predict the DNA-BPs by exploiting protein sequence comprehensively. However more explorations about amino acid properties are still needed in this direction and further work on interpreting these features and exploring mechanisms of protein-DNA interactions are underway.

## Competing interests

The authors declare that they have no competing interests.

## Authors’ contributions

HL conceived the study, reviewed and revised the manuscript. CZ developed the methods, performed the analysis and drafted the manuscript. JG supervised the whole project and participated in manuscript preparation. All authors have read and approved the final manuscript.

## Authors’ information

Correspondence and requests for reprints to:

Prof. Honglin Li

School of Pharmacy, East China University of Science and Technology 130 Mei Long Road, Shanghai 200237

Phone/Fax: +86-21-64250213

## Supplementary Material

Additional file 1 Complete list of PDB codes for DNAdset and DNAiset. Click here for file

Additional file 2 The IFS results for DNAdset. Click here for file

Additional file 3 The IFS results for DNAaset. Click here for file

Additional file 4 The IFS results and model evaluation for DNArset. Click here for file
