# Supplementary material for: An improved sequence based prediction protocol for DNA-binding proteins using SVM and comprehensive feature analysis
Source: BMC Bioinformatics. 2013 Mar 9;14:90. doi: 10.1186/1471-2105-14-90 (PMC3602657; doi:10.1186/1471-2105-14-90)
Supplement: Additional file 1 — Complete list of PDB codes for DNAdset and DNAiset. [file 1471-2105-14-90-S1.doc]

**(The fifth letter inicates protein chain number).**

**Table S1** Complete list of PDB codes for DNAdset

| **DNA-BPs** | | | | **non-DNA-binding proteins** | | | |
| --- | --- | --- | --- | --- | --- | --- | --- |
| 1A0AA | 1IF1A | 1RH6A | 2EZVA | 1A8EA | 1C52A | 1HXNA | 1PHPA |
| 1A02F | 1IG9A | 1RRQA | 2FIOA | 1A8PA | 1CA1A | 1HYPA | 1PHRA |
| 1A02N | 1IGNA | 1RXWA | 2FKCA | 1A8YA | 1CECA | 1IAEA | 1PHTA |
| 1A3QA | 1IU3C | 1RZRA | 2FLDB | 1A53A | 1CEMA | 1IDOA | 1PLCA |
| 1AKHA | 1J1VA | 1SA3A | 2G1PA | 1AACA | 1CFBA | 1IFCA | 1PMIA |
| 1AM9A | 1JB7A | 1SFUA | 2GB7A | 1ABEA | 1CHDA | 1INPA | 1PNEA |
| 1AZPA | 1JE8A | 1SKNP | 2GLIA | 1AC5A | 1CIYA | 1IOVA | 1POAA |
| 1B01A | 1JEYA | 1SVCP | 2H7GX | 1AEWA | 1CLCA | 1JDWA | 1POCA |
| 1B3TA | 1JEYB | 1SXPB | 2H27A | 1AH7A | 1CNVA | 1JERA | 1POTA |
| 1BC8C | 1JFIA | 1T2KD | 2HDCA | 1AHOA | 1COTA | 1KLOA | 1PPNA |
| 1BDTA | 1JFIB | 1TAUA | 2HDDA | 1AIRA | 1CPOA | 1KOEA | 1PRNA |
| 1BG1A | 1JJ4A | 1TF3A | 2HEOD | 1AL3A | 1CPQA | 1KPFA | 1PTFA |
| 1BHMA | 1JNMA | 1TQEQ | 2I13A | 1ALHA | 1CPTA | 1KTEA | 1PUCA |
| 1BL0A | 1JT0A | 1TROA | 2IBSA | 1ALUA | 1CSHA | 1KUHA | 1RA9A |
| 1BPYA | 1JX4A | 1TSRA | 2IHMA | 1ALYA | 1CSNA | 1LAMA | 1RB9A |
| 1C9BA | 1K3WA | 1TTUA | 2IHNA | 1AMFA | 1CTJA | 1LBUA | 1RCBA |
| 1CEZA | 1K4TA | 1U3EM | 2IIEA | 1AMKA | 1CTTA | 1LCLA | 1RECA |
| 1CF7A | 1KB4A | 1U8BA | 2IS2B | 1AMMA | 1CV8A | 1LEDA | 1RFSA |
| 1CF7B | 1KC6A | 1U78A | 2NTCA | 1AMPA | 1CVLA | 1LFOA | 1RHSA |
| 1CKTA | 1KU7A | 1UBDC | 2O4AA | 1AMXA | 1CYOA | 1LIDA | 1RIEA |
| 1CMAA | 1LLMC | 1UUTA | 2OAAA | 1AOAA | 1CZJA | 1LITA | 1RKDA |
| 1CW0A | 1LMB3 | 1V15A | 2ODIA | 1AOLA | 1DDTA | 1LKIA | 1RMGA |
| 1D02A | 1LQ1D | 1VASA | 2P0JA | 1AQBA | 1DFXA | 1LSTA | 1RSYA |
| 1D2IA | 1M3QA | 1W0UA | 2P5LC | 1ARBA | 1DHNA | 1LTMA | 1RZLA |
| 1D3UA | 1M6XC | 1WTEA | 2P6RA | 1ARUA | 1DHRA | 1MAIA | 1SBPA |
| 1DC1A | 1MDYA | 1X9NA | 2PYJA | 1ASHA | 1DINA | 1MATA | 1SEKA |
| 1DCTA | 1MNMA | 1XO0B | 2QHBA | 1ASSA | 1DOIA | 1MAZA | 1SFPA |
| 1DEWA | 1MNMC | 1XPXA | 2QNFA | 1AT0A | 1DPEA | 1MBAA | 1SKFA |
| 1DMUA | 1MOWD | 1XSDA | 2QSHA | 1ATGA | 1DRWA | 1MLAA | 1SMDA |
| 1DP7P | 1MTLA | 1Y6FA | 2R1JL | 1AUKA | 1DUNA | 1MOQA | 1SRAA |
| 1E3OC | 1MURA | 1YF3A | 2RBAA | 1AV4A | 1DXYA | 1MPPA | 1SURA |
| 1ECRA | 1NKPA | 1YUIA | 2RBFA | 1AXNA | 1EAFA | 1MRPA | 1SVBA |
| 1EFAA | 1NLWD | 1Z9CA | 2RGRA | 1AYLA | 1ECYA | 1MSKA | 1SVYA |
| 1EMHA | 1ODHA | 1Z63A | 2VJVA | 1B0BA | 1EDGA | 1MUPA | 1SYMA |
| 1EONA | 1OE4A | 1ZBLB | 2VLAA | 1B6AA | 1ESCA | 1NARA | 1TCAA |
| 1EQZA | 1OH5B | 1ZMEC | 2Z3XA | 1B51A | 1EZMA | 1NDHA | 1TDEA |
| 1EQZB | 1OMHA | 1ZS4A | 2ZHGA | 1BA3A | 1FCEA | 1NEUA | 1TENA |
| 1EQZC | 1ORNA | 1ZTWA | 3BKZA | 1BB9A | 1FDSA | 1NFPA | 1TFEA |
| 1EQZD | 1OUPA | 1ZX4A | 3BS1A | 1BD8A | 1FITA | 1NG1A | 1THVA |
| 1F4KB | 1OZJA | 2A3VA | 3BTXA | 1BDBA | 1FKJA | 1NIFA | 1TMLA |
| 1FIUA | 1P7DB | 2A66A | 3C2IA | 1BDOA | 1FMKA | 1NKRA | 1TMYA |
| 1FJLA | 1P8KZ | 2AORA | 3C25A | 1BEAA | 1FNCA | 1NLSA | 1TN3A |
| 1FOKA | 1P71A | 2AQ4A | 3CLCA | 1BEOA | 1FRBA | 1NNCA | 1TONA |
| 1GCCA | 1PNRA | 2AS5F | 3CLZA | 1BFDA | 1FUAA | 1NOXA | 1TRYA |
| 1GD2E | 1PP7U | 2BDPA | 3COQA | 1BG2A | 1FUSA | 1NPKA | 1TULA |
| 1GDTA | 1PUEE | 2BGWA | 3CROL | 1BG6A | 1G3PA | 1NSJA | 1UCHA |
| 1GT0D | 1PUFA | 2BNWA | 3DFXA | 1BGCA | 1GAIA | 1OBRA | 1UOKA |
| 1GU4A | 1PVIA | 2BOPA | 3HTSB | 1BHEA | 1GCAA | 1OPRA | 1USHA |
| 1GXPA | 1PYIA | 2BSQE | 3L2CA | 1BHPA | 1GENA | 1OPSA | 1UTGA |
| 1H0MA | 1QBJB | 2BZFA | 6CROA | 1BJ7A | 1GKYA | 1OPYA | 1VLSA |
| 1H6FA | 1QNAA | 2C5RA | 6PAXA | 1BK0A | 1GNDA | 1OSAA | 1WL9A |
| 1H9DA | 1QRVA | 2C9LZ |  | 1BOBA | 1GOFA | 1OYCA |  |
| 1H89C | 1QZHA | 2CGPA |  | 1BPIA | 1GPRA | 1PBEA |  |
| 1HCRA | 1R0AA | 2DP6A |  | 1BQKA | 1GSAA | 1PBVA |  |
| 1HLOA | 1R2ZA | 2DPIA |  | 1BR9A | 1HCZA | 1PDAA |  |
| 1HLVA | 1R7MA | 2DRPA |  | 1BS9A | 1HFCA | 1PDOA |  |
| 1HWTC | 1R8DA | 2E1CA |  | 1BTNA | 1HKAA | 1PEAA |  |
| 1I3JA | 1R8EA | 2E52A |  | 1BV1A | 1HOEA | 1PGSA |  |
| 1IAWA | 1R71A | 2ER8A |  | 1BX7A | 1HPMA | 1PHDA |  |
| 1IC8A | 1REPC | 2EX5A |  | 1BYBA | 1HTPA | 1PHKA |  |

**Table S2** Complete list of PDB codes for DNAiset

| **DNA-BPs** | | | | **non-DNA-binding proteins** | | | |
| --- | --- | --- | --- | --- | --- | --- | --- |
| 3AU6A | 3T1HI | 3UVFA | 4DQYB | 1A12A | 1Q74A | 2IAPA | 3BB0A |
| 3QOQA | 3T1HJ | 3V4RA | 4DQYC | 1BCH1 | 1QL3A | 2J9FA | 3BDZA |
| 3QWSA | 3T1HK | 3V6HA | 4E5ZB | 1BF6A | 1QZ0A | 2JINA | 3C1JA |
| 3RADA | 3T1HM | 3V7EA | 4E9FA | 1BYPA | 1R8ME | 2NS9A | 3CGIA |
| 3RADC | 3T1HN | 3V20A | 4E54A | 1C7JA | 1RFXA | 2NT3A | 3CIFA |
| 3REHA | 3T1HO | 3V79C | 4ENJA | 1CHMA | 1RQWA | 2O2GA | 3CZVA |
| 3REHB | 3T1HP | 3V79K | 4ER8A | 1CMNA | 1RWIB | 2O6XA | 3D0KA |
| 3REHC | 3T1HQ | 3V79M | 4ESJA | 1CUHA | 1SCTB | 2O7IA | 3D1RA |
| 3RI4A | 3T1HR | 3VD0A | 4EUWA | 1CZYA | 1TEJB | 2OFXA | 3DR3A |
| 3RJEA | 3T1HS | 3VDYA | 4F1NA | 1D7EA | 1UAIA | 2OQAA | 3DV9A |
| 3RKQA | 3T1HT | 3VKEA | 4F6MA | 1DHIA | 1UHAA | 2OQYA | 3E3UA |
| 3RMPA | 3TQ1A | 3VOKA | 4FCYA | 1DQEA | 1UJ0A | 2P35A | 3EJVA |
| 3RN2A | 3TWMA | 3ZQCA | 4FVUA | 1DXKA | 1UJMA | 2P3JA | 3EMHA |
| 3RZGA | 3U5ZA | 4A8FA | 4FX4A | 1E0WA | 1UWLA | 2PRVA | 3ENUA |
| 3S8QA | 3U5ZB | 4A75A | 4G7HA | 1E2XA | 1V3IA | 2Q5XA | 3EOFA |
| 3S58A | 3U5ZF | 4AAEA | 4G7HC | 1E30A | 1VAJA | 2Q66A | 3EWDA |
| 3SJMA | 3U6YA | 4AQYL | 4G7HD | 1E39A | 1VDWA | 2QECA | 3FIRA |
| 3SSCA | 3U44A | 4ASOA | 4G7HE | 1E6FA | 1VLCA | 2QIQA | 3G5JA |
| 3SWMA | 3U44B | 4B3GA | 4G7HF | 1E6WA | 1WSRA | 2QKFA | 3GBXA |
| 3T1HB | 3UBTA | 4BACA | 4G92A | 1EW4A | 1XA3A | 2QM0A | 3GHJA |
| 3T1HC | 3UDGA | 4DB4A | 4G92B | 1FAZA | 1XFIA | 2QM6A | 3GWBA |
| 3T1HD | 3UGMA | 4DH9Y | 4G92C | 1FPZA | 1XH9A | 2QNDA | 3GY1A |
| 3T1HE | 3UIQA | 4DIHH | 4GFHA | 1FY3A | 1XKWA | 2QQRA | 3GZRA |
| 3T1HF | 3ULPA | 4DM0A | 4GZ0A | 1G8KB | 1XMZA | 2QRJA | 3H36A |
| 3T1HG | 3UPQA | 4DQIA | 4GZNC | 1GPIA | 1Y0BA | 2QSIA | 3H3JA |
| 3T1HH | 3UT9D | 4DQYA | 4H5QA | 1GQGA | 1YCLA | 2R1BA | 3H5QA |
|  |  |  |  | 1GWTA | 1YNVX | 2RAUA | 3H8TA |
|  |  |  |  | 1GXJA | 1YPQA | 2RB8A | 3H9CA |
|  |  |  |  | 1H0HB | 1YU0A | 2RBDA | 3INGA |
|  |  |  |  | 1H2GB | 1YUKA | 2RK6A | 3K2CA |
|  |  |  |  | 1H6WA | 1Z3EB | 2RLCA | 3K3KA |
|  |  |  |  | 1HQGA | 1Z82A | 2V4CA | 3K8GA |
|  |  |  |  | 1I9YA | 1ZCJA | 2V5IA | 3KFFA |
|  |  |  |  | 1J8MF | 1ZDQA | 2V9MA | 3LIDA |
|  |  |  |  | 1JLJA | 1ZE3D | 2VLBA | 3LX4A |
|  |  |  |  | 1JNRA | 1ZHVA | 2VLPB | 3M12A |
|  |  |  |  | 1KTBA | 1ZLHB | 2VR3A | 3M1MA |
|  |  |  |  | 1KZ1A | 2A33A | 2W87A | 3M7OA |
|  |  |  |  | 1LRIA | 2AD6A | 2W8TA | 3MCXA |
|  |  |  |  | 1LT4A | 2AHEA | 2WGVA | 3ME7A |
|  |  |  |  | 1LVMA | 2B1LA | 2WL5A | 3MR1A |
|  |  |  |  | 1NIJA | 2C15A | 2Y88A | 3MSUA |
|  |  |  |  | 1O2DA | 2CB9A | 2Z25A | 3N20A |
|  |  |  |  | 1OAIA | 2CN3A | 2Z58B | 3N2BA |
|  |  |  |  | 1OCBA | 2CWLA | 2ZU1A | 3NJCA |
|  |  |  |  | 1ODZA | 2EX4A | 2ZZ3A | 3NK6A |
|  |  |  |  | 1OJQA | 2FHLA | 3A75B | 3NXDC |
|  |  |  |  | 1OX3A | 2FW6A | 3A8GA | 3NZNA |
|  |  |  |  | 1P0WA | 2G8FA | 3AHYA | 3OCCA |
|  |  |  |  | 1PAHA | 2GAIA | 3APAA | 3OIRA |
|  |  |  |  | 1PC5A | 2GAKA | 3B5NC | 3OYYA |
|  |  |  |  | 1PF3A | 2GJPA | 3B7AA | 3QD5A |
|  |  |  |  | 1PGTA | 2GRRA | 3B7CA | 3R0NA |
